# Supplementary material for: The interactome of intact mitochondria by cross-linking mass spectrometry provides evidence for coexisting respiratory supercomplexes
Source: Mol Cell Proteomics. 2017 Dec 8;17(2):216–32. doi: 10.1074/mcp.RA117.000470 (PMC5795388; doi:10.1074/mcp.RA117.000470)
Supplement: Supplemental Data [file supp_RA117.000470_133922_0_supp_23978_fzffwf.zip › spectra_annotation/mito_spectra_annotation/306-1-4-1-3-1.pdf]

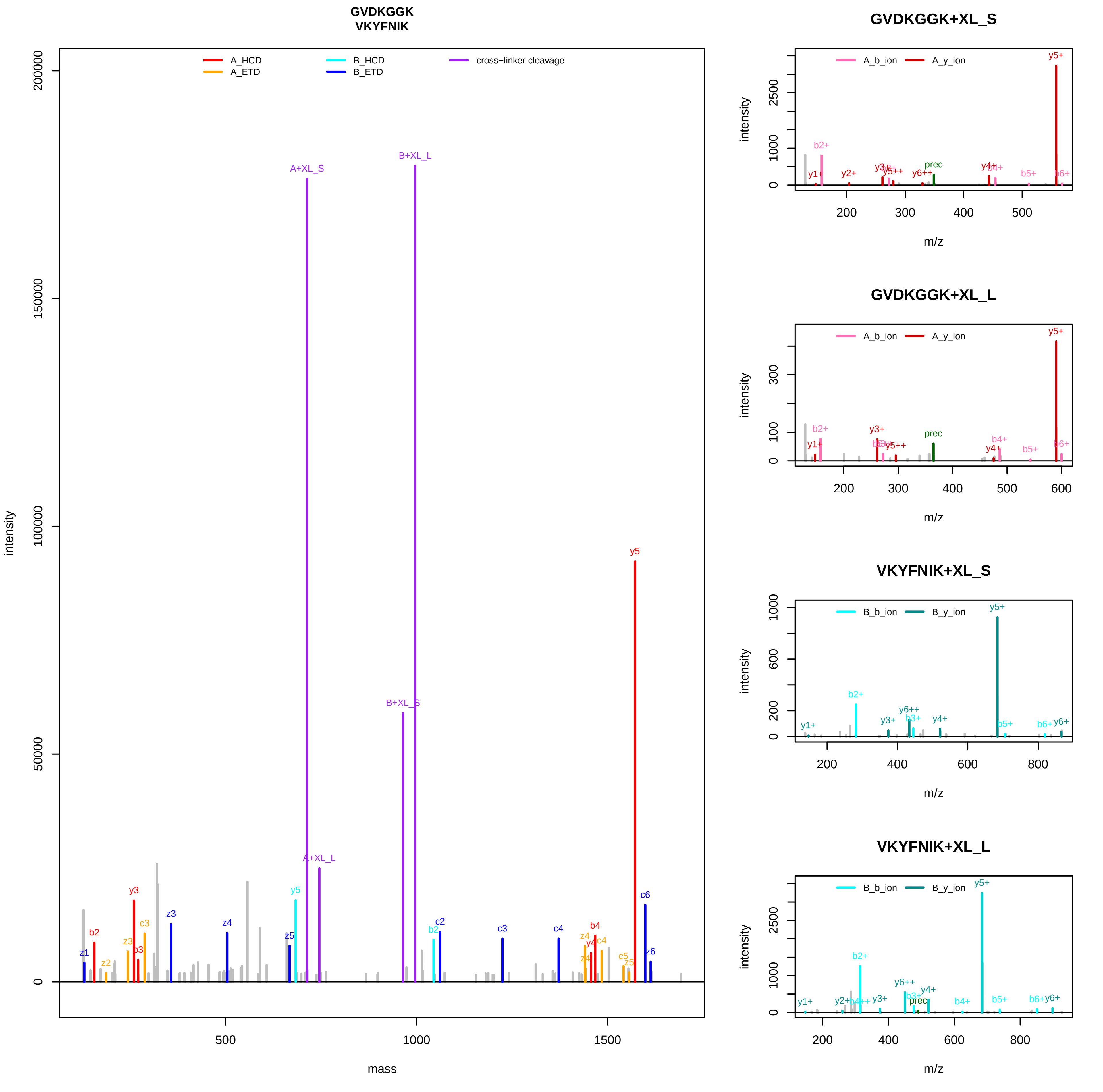

GVDKGGK
VKYFNIK
A_HCD
A_ETD
B_HCD
B_ETD
cross−linker cleavage
A+XL_S
B+XL_L
B+XL_S
A+XL_L
y5
y3
b2
b3
b4
y4
z1
z2
z3
z4
z5
z6
c2
c3
c4
c5
c6
intensity
mass
0
50000
100000
150000
200000
500
1000
1500
GVDKGGK+XL_S
GVDKGGK+XL_L
A_b_ion
A_y_ion
VKYFNIK+XL_S
VKYFNIK+XL_L
B_b_ion
B_y_ion
prec
b2+
y1+
y2+
y3+
y4+
y5+
y5++
y6++
y6+
b3+
b4+
b4++
b5+
b6+
m/z
